# Smart polymers driven by multiple and tunable hydrogen bonds for intact phosphoprotein enrichment

**DOI:** 10.1080/14686996.2019.1643259

**Published:** 2019-07-15

**Authors:** Xiaofei Zhang, Qi Lu, Cheng Chen, Xiuling Li, Guangyan Qing, Taolei Sun, Xinmiao Liang

**Affiliations:** a Key Laboratory of Separation Science for Analytical Chemistry, Dalian Institute of Chemical Physics, Chinese Academy of Sciences, Dalian, P. R. China; b State Key Laboratory of Advanced Technology for Materials Synthesis and Processing, Wuhan University of Technology, Wuhan, P. R. China; c Research & Development Center, Jushi Group. Co., P. R. China

**Keywords:** Phosphoprotein, enrichment, hydrogen bond, 30 Bio-inspired and biomedical materials, 212 Surface and interfaces

## Abstract

Separation of phosphoproteins is essential for understanding their vital roles in biological processes and pathology. Transition metal-based receptors and antibodies, the routinely used materials for phosphoproteins enrichment, both suffer from low sensitivity, low recovery and coverage. In this work, a novel smart copolymer material was synthesized by modifying porous silica gel with a poly[(*N*-isopropylacrylamide-*co*-4-(3-acryloylthioureido) benzoic acid)0.35] (denoted as NIPAAm-*co*-ATBA0.35@SiO_2_). Driven by the hydrogen bonds complexation of ATBA monomers with phosphate groups, the copolymer-modified surface exhibited a remarkable adsorption toward native α-casein (a model phosphoprotein), accompanied with signiﬁcant changes in surface viscoelasticity and roughness. Moreover, this adsorption was tunable and critically dependent on the polarity of carrier solvent. Beneﬁting from these features, selective enrichment of phosphoprotein was obtained using NIPAAm-*co*-ATBA_0.35_@SiO_2_ under a dispersive solid-phase extraction (dSPE) mode. This result displays a good potential of smart polymeric materials in phosphoprotein enrichment, which may facilitate top-down phosphoproteomics studies.

## GRAPHICAL ABSTRACT

**Figure:**
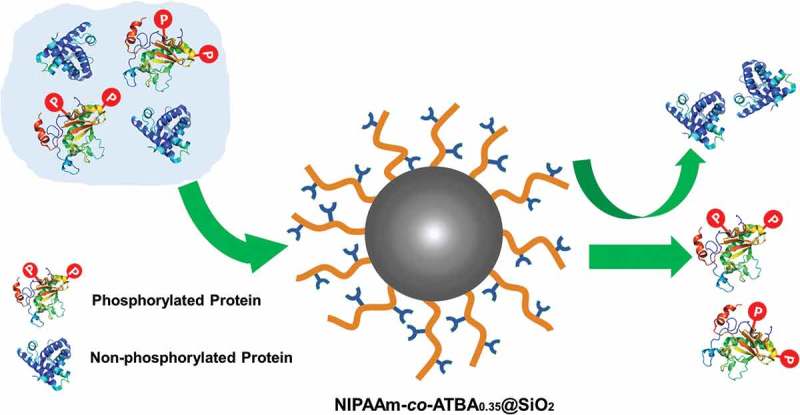

## 1. Introduction

Protein phosphorylation is a specific and reversible post-translational modification, which regulates numerous biological events, such as signal transduction, gene expression, and the cell cycle [1,2]. Substantial studies have revealed the close relationships between abnormal protein phosphorylation and aberrant protein functions, which subsequently lead to many critical diseases (i.e. cancers [3,4] and neurodegenerative diseases [5]). Thus, phosphorylated proteins have attracted increasing interest of scientists working in biology, pathology and therapeutics.

Nowadays, analysis of protein phosphorylation adopts either bottom-up or top-down strategy. The bottom-up strategy comprises digestion of proteins, enrichment of phosphopeptides from peptide pool, mass spectrometry (MS) analysis and database searching [6]. However, this process might result in ambiguous characterization of alternative splice forms [7], endogenous protein cleavages, and intact protein isoforms [8]. Top-down phosphoproteins can solve these problems.

In the last decade, several materials have been developed to enrich phosphoproteins and to reduce the complexity of bio-samples [9–12]. These materials mainly include species based on coordination interactions and multiple hydrogen bonding. The coordination interaction-based materials take advantages of the strong affinity between phosphate groups and transition metal cations, representative with immobilized metal affinity chromatography (IMAC) materials [13–15] and TiO_2_ [16,17]. For example, Zn^2+^-immobilized superparamagnetic nanoparticles with multivalent ligand molecules were developed and used for phosphoproteins enrichment [13]. The self-assembled TiO_2_ nanocrystal clusters [16] and the flowerlike microspheres with hierarchical porous TiO_2_ [17] demonstrated good performance in phosphoproteins enrichment. However, the affinity between these materials and phosphoproteins is too strong to be regulated, leading to the low recovery of phosphoproteins. The antibody-based on multiple hydrogen bonding could specifically bind to phosphoproteins [18,19]. However, the commercial available antibodies only work for proteins with phosphorylated tyrosine residues [20]. The overwhelming majority (99%) [21] of phosphoproteins with phosphorylated serine and threonine residues can hardly be enriched with immunoprecipitation (IP) because of their smaller antibody-antigenic determinants [22]. To mimic the multiple hydrogen bonds in nature, magnetic nanospheres were grafted with polymer brushes containing abundant grandly groups [23]. The novel material possesses the specific capture capacity of phosphoproteins rely on the more stable and more extensive functional affinity sites [23,24]. Although the progress has been made in the enrichment materials of phosphoproteins, the gap between artificial polymer and native ones needs to be filled. Therefore, developing a high specificity, high sensitivity and more effective material for phosphoproteins enrichment is urgently needed.

**Scheme 1.**
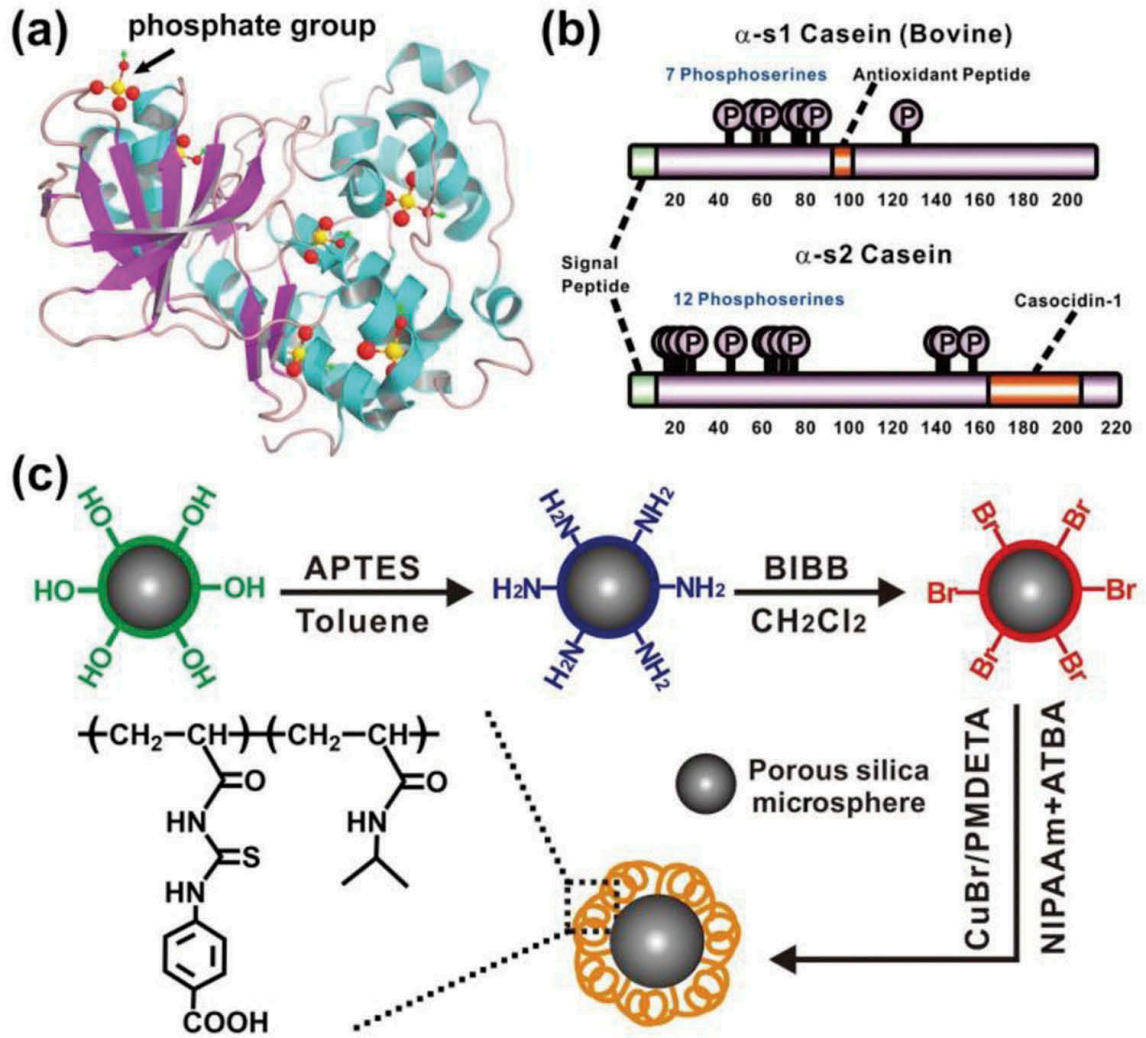
(a) 3D schematic of a common phosphoprotein, in which most of the phosphate groups are concealed in the amino acid network. (b) Primary structure of α-s1 casein and α-s2 casein (main components of α-casein sample used in this work), containing sequence information, especially phosphorylated sites of these two proteins. (c) Synthesis route of the smart copolymer-modified silica gel (NIPAAm-*co*-ATBA_0.35_@SiO_2_).

Here we report a smart polymer-based material for phosphoprotein enrichment, which is constructed by immobilizing a poly[(*N*-isopropylacrylamide-*co*-4-(3-acryloyl-thioureido) benzoic acid)_0.35_] (denoted as NIPAAm-*co*-ATBA_0.35_) onto the surface of porous silica gel. In the copolymer, ATBA as the core recognition unit displays strong affinity toward α-casein (containing two phosphoproteins―α-s1 casein and α-s2 casein, and phosphorylated sites are shown in Scheme 1(b). The flexible poly (NIPAAm) affords a tunable hydrogen bonds network, capable of intelligently modulating the movement of polymer chains in response to the adsorption of α-casein [25], which will in turn remarkably inﬂuence the binding and release of the guest proteins [26,27]. In addition, the porous silica gel with large surface area allows high grafting density of the smart copolymer. Based on this design, the copolymer-modified silica gel (NIPAAm-*co*-ATBA_0.35_@SiO_2_) was successfully applied into the separation of α-casein and bovine serum albumin (BSA, a commonly used non-modified protein).

## 2. Experimental details

### 2.1. Chemicals for material synthesis and characterization

Porous amino-modified silica gel (diameter 5 μm, average pore size 350 Å) was provided by ACCHROM (a Chinese company in Beijing). ATBA was synthesized through a two-step coupling reaction [27]. *N*-isopropyl acrylamides (99%, Sigma-Aldrich) was recrystallized in n-hexane for three times before polymerization [28]. Acryloyl chloride (97%), potassium thiocyanate (KSCN, 99%), 4-aminobenzoic acid (3-aminopropyl) triethoxysilane (APTES, 98%), 2-bromoisobutyryl bromide (BIBB, 99%), pyridine (99%), *N,N,N*’,*N*’’,*N*’’-pentamethyldiethylenetriamine (PMDETA, 99%), copper (I) bromide (CuBr, 98%), tris (hydroxy-methyl) aminomethane (Tris, 98%), bovine serum albumin (BSA), α-casein were purchased from Sigma-Aldrich. Deuterated water (D_2_O, 99%) was purchased from Sigma-Aldrich (China). Toluene, acetonitrile (CH_3_CN), ethanol, acetone, dichloromethane, *N, N*-dimethylformamide (DMF), dimethylsulfoxide (DMSO), sodium hydroxide (NaOH) and hydrochloric acid (HCl) were ordered from Merck (Darmstadt, Germany) and used as received. Trifluoroacetic acid (TFA) was obtained from TEDIA (Fairfield, USA). Double distilled water (18.2 MΩ·cm, Milli-Q system, Bedford, MA, USA) was used in the experiments.

### 2.2. Demonstration of the interaction between ATBA and α-casein

#### 2.2.1. Fluorescence titration experiment

To investigate the binding properties of ATBA monomer with α-casein or BSA, fluorescence titration experiments were performed, which is a typical and widely adopted method for calculating the association constant (*K*a) in host-guest chemistry [29]. Host proteins were prepared as stock solutions in Tris−HCl buffer solution (1 m·mol^‒1^, pH 7.4) at 20°C; the protein concentrations were 60 μg·mL^‒1^ for α-casein and 90 μg·mL^‒1^ for BSA. Guest ATBA were prepared to 0.01 and 0.10 mol·L^‒1^ of stock solution in DMSO. The work solutions were prepared by adding diﬀerent volumes of guest solution and the same amount of protein host (1 mL) to a series of test tubes, followed by dilution to 3.00 mL by Tris-HCl buﬀer solution (1 m·mol^‒1^, pH 7.4). After being shaken for 0.5 min, the work solutions were measured immediately at 20°C using a Perkin Elmer LS-55 spectrometer (Perkin Elmer, Inc., USA, excitation wavelength: 280 nm). The *K*a value between ATBA and protein was obtained through a nonlinear ﬁtting calculation according to the ﬂuorescence intensity changes in the maximum emission peak (340 nm).

#### 2.2.2. Circular dichroism (CD) titration experiment

For the exploration of the protein conformational changes after interacting with ATBA, CD measurement [30] was conducted on a J-1500 CD spectrophotometer (JASCO, Japan). Host proteins were prepared as stock solutions in Tris-HCl buﬀer solution (1 m·mol^‒1^, pH 7.4) at 20°C; the concentration was 450 μg·mL^‒1^ for α-casein and 210 μg·mL^‒1^ for BSA. Guest ATBA were prepared to 1 mg·L^‒1^ of stock solution in Tris-HCl buﬀer solution (1 m·mol^‒1^, pH 7.4). The work solutions were prepared by adding different volumes of ATBA solution to a series of test tubes, and then 1 mL stock solution of host protein was added into each test tube, followed by dilution to 3.00 mL by the Tris-HCl buﬀer solution (1 mmol·L^‒1^, pH 7.4). Then, the sample was transferred into a cuvette (volume: 3 mL). CD spectra were recorded in a range of 200 to 260 nm at a scan rate of 0.2 nm·s^‒1^ at 20°C. Raw data were processed by the subtraction of the solvent spectra and then manipulated by smoothing the curves once.

#### 2.2.3. Bio-attenuated total reﬂectance Fourier transform infrared spectroscopy (Bio-ATR-FTIR) titration experiment

The infrared spectra were recorded on a Bruker Vertex 80v FTIR spectrometer (Bruker, Germany) in Bio-ATR cell II accessory. All samples were dissolved in 16 μL of D_2_O. For reach sample, the concentrations (150 μg·mL^‒1^ proteins and 0.5 or 1.0 equivalent of ATBA) and total volume (16 μL) were strictly controlled. For each measurement, the equipment remained in standby mode for 10 min to ensure the equilibrium of temperature (20°C) prior to the test, and all the spectra of samples were obtained through 1200 scans subtracting the D_2_O background at a 4 cm^−1^ resolution. Before each measurement, the Bio-ATR cell was cleaned with distilled water and ethanol, respectively. Then, it was suﬃciently dried under a nitrogen gas ﬂow.

#### 2.2.4. Experiment of quartz−crystal microbalance with dissipation (QCM-D)

First, Au-coated quartz−crystal (QC) resonators with an intrinsic frequency (*F*_0_) of 5 MHz (purchased from Q-Sense Corp., Sweden) were modified with a NIPAAm-*co*-ATBA polymeric film [27]. Then all QCM-D measurements were performed using these copolymer-modified QC resonators (at 20°C) on a Q-Sense E4 system (Q-Sense Corp., Sweden). Prior to binding assays between the model proteins (i.e. α-casein and BSA) and the copolymer, QCM channels and tubes were washed carefully with distilled water and dried under a ﬂow of nitrogen gas, followed by installation of the functionalized QC resonator into a ﬂow-cell for frequency and dissipation measurements. After stabilization of the fundamental resonance frequency with pure water and buffer solution (CH_3_CN/H_2_O mixture with a volume ratio of 10:90 or 30:70, pH 7.4) at 20°C, protein solution (100 μg·mL^−1^) was pumped into the ﬂow-cell by a peristaltic pump at a constant speed of 100 μL·min^−1^. All of the time-dependent frequency and dissipation curves were recorded with Q-Sense software and analysed by Q-Tools [31].

#### 2.2.5. Atomic force microscopy (AFM) and surface contact angle (CA) measurement

AFM measurement was performed using a Multimode 8 AFM instrument (Bruker, Germany). For discernment of the changes in surface morphology and roughness of the copolymer ﬁlm before and after protein adsorption, the NIPAAm-*co*-ATBA-modiﬁed QC resonator was treated by a solution of protein (100 μg·mL^−1^ α-casein or BSA in CH_3_CN/H_2_O mixture with a volume ratio of 30:70) for 10 min at 20°C, which was followed by AFM measurements in the scan mode under ambient condition [32]. Then, the static CA was recorded for each QC resonator using the sessile drop method (at ambient atmosphere and a constant temperature of 20°C) and pure water as a solvent [33]. Each measurement was repeated in triplicate to ensure the reliability of data.

### 2.3. Synthesis of NIPAAm-co-ATBA_0.35_@SiO_2_

NIPAAm-*co*-ATBA_0.35_@SiO_2_ was prepared through a typical surface-initiated atom transfer radical polymerization (SI-ATRP) according to the literature [34]. The rationality of the copolymer design has been validated in our previous work [27]. First, the amino-modified silica gel was orderly cleaned with toluene (100 mL), and dichloromethane (100 mL), and then was dispersed in dichloromethane (40 mL) containing 0.5% v/v pyridine. After the mixture was stirred at 0°C for 10 min, the polymerization initiator BIBB (0.2 mL) was added dropwise into the reactants, and the mixture was stirred for 1 h at this temperature and then at room temperature for 10 h. The product was vacuum filtered, cleaned with dichloromethane and dried in vacuum oven (40°C, 12 h). The obtained silica gel was dispersed in a degassed solution of NIPAAm (0.45 g, 4 mmol), ATBA (0.50 g, 2 mmol) in a mixture of 50 ml DMF containing CuBr (0.032 g, 0.23 mmol). After strict removal of oxygen, PMDETA (0.12 mL) was injected into the reactants immediately and then stirred for 6 h, at 60°C. The product was vacuum filtered, orderly cleaned with DMF, water and ethanol, and then was dried in vacuum oven (40°C, 12 h).

### 2.4. Characterization of NIPAAm-co-ATBA_0.35_@SiO_2_

Morphology and composition analysis of prepared NIPAAm-*co*-ATBA_0.35_@SiO_2_ materials was performed through scanning electron microscopy (SEM), nitrogen isothermal adsorption, thermal gravimetric analysis (TGA) and organic element analysis. High-resolution SEM was carried out on a Hitachi S4800 (Hitachi Corp., Tokyo, Japan). Nitrogen isothermal adsorption was recorded on a Micromeritics Tri Star II 3020 nitrogen adsorption-desorption apparatus (Micromeritics Instrument Corp., GA, USA). Zeta-potential of NIPAAm-*co*-ATBA_0.35_@SiO_2_ was measured using a Zetasizer Nano ZS (Malvern Instruments Ltd., UK). Aqueous solution with different pH values were adjusted by adding NaOH or HCl; the sample concentration was 100 μg·mL^‒1^ for NIPAAm-*co*-ATBA_0.35_@SiO_2_ and 40 μg·mL^‒1^for proteins (i.e. α-casein and BSA).

### 2.5. Selective enrichment of phosphoproteins with NIPAAm-co-ATBA_0.35_@SiO_2_

#### 2.5.1. Selective enrichment of α-casein from the mixture of model proteins

Enrichment of α-casein was carried out under a dispersive solid-phase extraction (dSPE) mode at room temperature. The model samples were α-casein and BSA. The mixture of BSA and α-casein at mass ratio of 1:5 was dissolved with CH_3_CN/H_2_O (30:70, v/v) and incubated with NIPAAm-*co*-ATBA_0.35_@SiO_2_ in Eppendorf tubes (1.5 mL). After incubation for 30 min, the supernatant was collected. The resulting pellet was washed twice with 100 μL of CH_3_CN/H_2_O (30:70, v/v). The bound proteins were eluted with 80 μL of CH_3_CN/H_2_O/TFA (10:89:1, v/v).

Proteins were characterized with Waters 2695 HPLC coupled to Waters 2998 photodiode array detector (PDA) (Waters, Milford, MA, USA). The HPLC column was BEH (5 μm, 300 Å, 150 mm × 4.6 mm inner diameter) obtained from Waters (Milford, MA, USA). The mobile phases were 0.1% TFA aqueous solution (A) and 100% CH_3_CN containing 0.1% TFA (B). The gradient was 30–50% B in 20 min. The flow rate was 1.0 mL·min^‒1^. The detector wavelength is 281 nm.

#### 2.5.2. Selective enrichment of phosphoproteins from defatted milk

Enrichment of phosphoproteins was carried out under a dSPE mode at room temperature. The defatted milk was firstly precipitated with acetone (at 1:4 in volume, −20^o^C, 4 h). Subsequently, the precipitate was redissolve with CH_3_CN/H_2_O (30:70, v/v) and incubated with NIPAAm-*co*-ATBA_0.35_@SiO_2_ for 30 min. After centrifugation, the supernatant was collected. The resulting pellet was washed twice with 500 μL of CH_3_CN/H_2_O (30:70, v/v). The bound proteins were eluted with 20 μL of CH_3_CN/H_2_O/TFA (10:89:1, v/v).

The detection method was the same as above.

## 3. Results and discussion

### 3.1. Complexation of ATBA with model proteins

**Figure 1.**
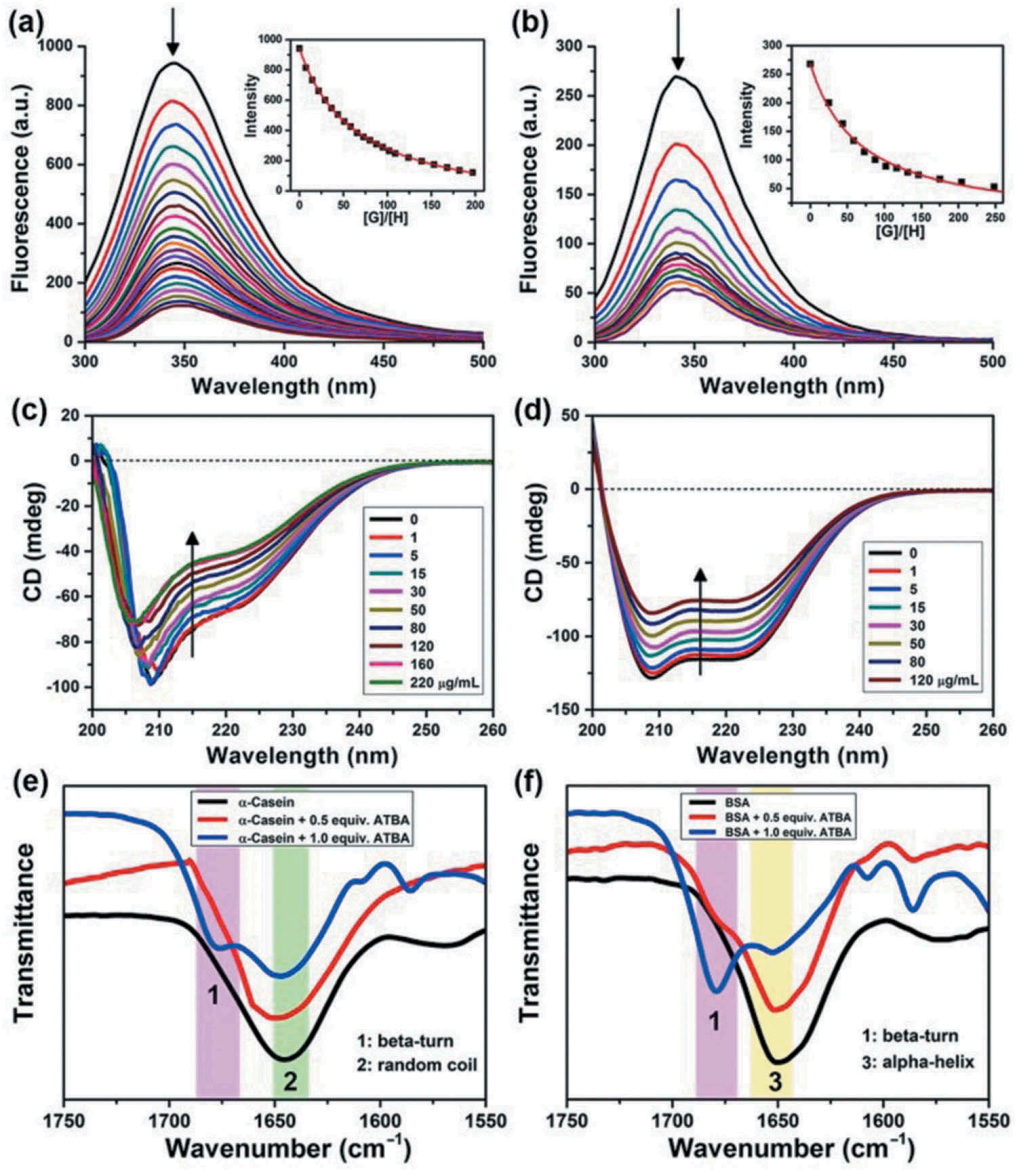
(a,b) Fluorescence spectra of α-casein (a) or BSA (b) with the addition of various equivalents of ATBA monomers in Tris-HCl buffer solution (1 mmol·L^‒1^) at pH 7.4 and 20°C; the protein concentration was 20 μg·mL^‒1^ or 30 μg·mL^‒1^ for α-casein or BSA, respectively. (c,d) Circular dichroism spectra of α-casein (c) or BSA (d) with the addition of various amounts of ATBA in Tris-HCl buffer solution (1 mmol·L^‒1^) at pH 7.4 and 20°C; the protein concentration was 150 μg·mL^‒1^ or 70 μg·mL^‒1^ for α-casein or BSA, respectively. (e,f) Representative FTIR spectra of the amide I band of α-casein (e) and BSA (f) after interaction with ATBA in D_2_O at 20°C (the colour bands in the figure illustrate three characteristic peaks corresponding to the secondary structures of the proteins, 1: ß -turn, 2: random coil, 3: α-helix).

To design the functional copolymer, the binding capacity of ATBA monomer with model proteins (i.e. α-casein and BSA) was investigated by ﬂuorescent titration [29], CD titration experiment [30], and Bio-ATR-FTIR titration experiment. As shown in Figure 1(a), a sharp decrease in ﬂuorescence intensity (at 340 nm) of α-casein was observed upon the additions of various equivalents of ATBA. It was the fluorescence quenching caused by the binding of ATBA monomer and α-casein. Subsequently, a nonlinear ﬁtting calculation according to the ﬂuorescence intensity changes was applied to obtain the association constant. In Figure 1(a) inset, [G]/[H] is an abbreviation of the molar ratio of guest ATBA to host α-casein. The red lines are nonlinear-fitted curves. The nonlinear calculation equation is listed as below:

$$\begin{array}{rl} & F=F_{0}+\frac{F_{\lim}-F_{0}}{2C_{0}}\{C_{H}\;+\;C_{G}\;+\;1/K_{ass}\\ & \;\;\;\;\;\;\;\;\;\;\;-[(C_{H}\;+\;C_{G}\;+\;1/K_{ass}{)}^{2}-4\;C_{H}\;C_{G}{]}^{1/2}\} \end{array}$$

where *F* represents the fluorescence intensity, *F*_0_ and *F*_lim_ are the initial and ultimate fluorescence intensity, respectively. *C*_H_ and *C*_G_ are the corresponding concentrations of host α-casein and guest ATBA. *C*_0_ is the initial concentration of host α-casein. *K*_ass_ is the association constant.

Through the nonlinear ﬁtting, an association constant (*K*_ass_) of 19,700 ± 500 L·mol^‒1^ was obtained. By comparison, the fluorescence intensity was measured when ATBA interacted with BSA (Figure 1(b)), and the corresponding *K*_ass_ was calculated (Figure 1(b) inset) as 7580 ± 860 L·mol^‒1^. These results implied the stronger binding affinity of ATBA toward phosphoprotein (α-casein) than non-phosphoprotein (BSA). In our previous work, we have verified the existence of hydrogen bonding between ATBA and phosphates [27]. Although there is an electrostatic repulsion between the negatively charged carboxyl groups of ATBA and the phosphates of α-casein at pH 7.4, we speculate that hydrogen bonding interaction was the main driving force for the complexation between ATBA and phosphates.

This remarkable complexation of ATBA with α-casein or BSA was further confirmed by CD titration experiment. Figure 1(c,d) displays the far UV CD spectra of α-casein and BSA upon the addition of different equivalents of ATBA. The data indicated that α-casein had a high content of α-helix and random coil in its native state at pH 7.4 [35], while BSA mainly had α-helix at this condition. Moreover, the native conformation of both the two proteins gradually changed when they interacted with ATBA, reflecting as a remarkable decrease in CD intensity of the two proteins. Then, the conformational changes were further verified by the FTIR experiment in a Bio-ATR mode, as shown in Figure 1(e,f). For α-casein, the signals of random coil structure (at 1645 cm^‒1^) decreased with the additions of ATBA, accompanied with the appearance of a new peak (at 1678 cm^‒1^), corresponding to β-turn structure. By comparison, the α-helix signal of BSA at 1650 cm^‒1^ obviously decreased when BSA interacted with ATBA. Meanwhile, a new peak around 1679 cm^‒1^ was also observed, which indicated the appearance of β-turn structure in BSA. In the above results, the changes in CD intensities and Bio-ATR signals were attributed to the conformational changes of the model proteins. The binding with ATBA monomer might incur the conformational changes of proteins. Therefore, the above results also implied the strong and distinct binding affinity of ATBA monomer toward the model proteins.

### 3.2. Adsorption of model proteins on NIPAAm-co-ATBA film

**Figure 2.**
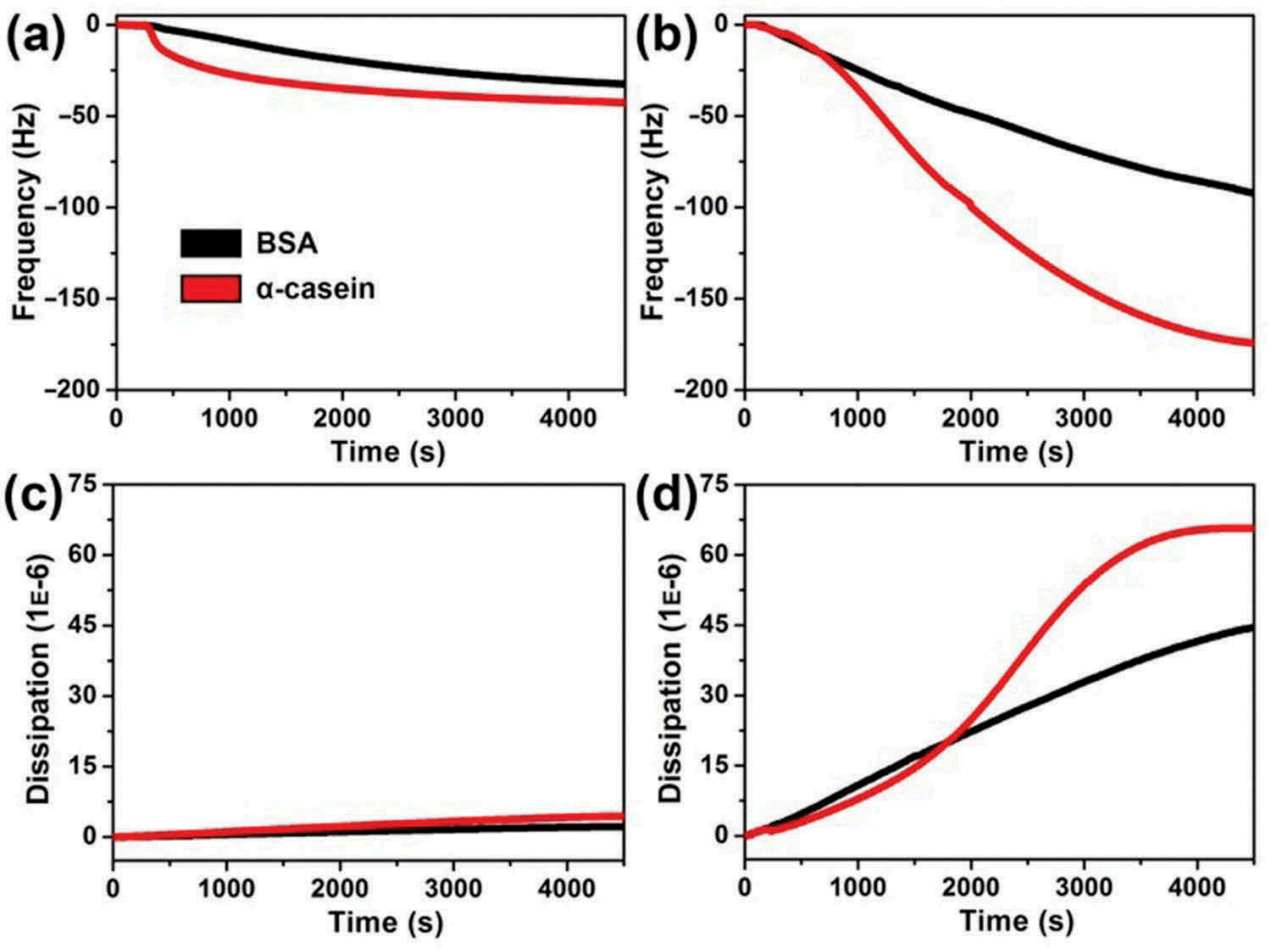
Time dependence of frequency (a,b) or dissipation (c,d) change curves during adsorption of α-casein or BSA (100 μg·mL^−1^) on the NIPAAm-*co*-ATBA modiﬁed QC resonator surfaces at 20°C; the carrier solvent was CH_3_CN/H_2_O mixture (pH 7.4) with a volume ratio of 10:90 (a,c) or 30:70 (b,d).

For improvement of this binding affinity of ATBA toward α-casein, and further amplification of the discrimination capacity from BSA, ATBA was copolymerized with NIPAAm through the SI-ATRP method, generating a NIPAAm*-co*-ATBA on the surface of an Au-coated QC resonator. Subsequently, dynamic adsorption behaviours of the model proteins on the NIPAAm*-co*-ATBA polymeric surface were monitored by QCM-D experiments. QCM-D simultaneously recorded the real-time variation in resonance frequency (Δ*f*) and energy dissipation (Δ*D*) when the mass adsorbed on an oscillated piezoelectric crystal changes [31]. As shown in Figure 2(a), the unbiased adsorption of α-casein and BSA only induced a slight decrease in resonance frequency (Δ*f* was less than –50 Hz), when the carrier solvent was CH_3_CN/H_2_O mixture with a volume ratio of 10:90. Interestingly, when the CH_3_CN ratio (v/v) was increased to 30%, α-casein displayed a remarkably accelerated adsorption on the NIPAAm-*co*-ATBA surface with a maximal Δ*f* of −172 Hz after 65 min (Figure 2(b)), corresponding to an adsorption quantity of 1.03 μg cm^−2^ according to the Sauerbrey equation [36]. By comparison, the frequency change caused by BSA was only −90 Hz at the same condition. The acetonitrile was used to reduce the nonspecific adsorption caused by some extent hydrophobic interactions of our adsorbent with hydrophobic proteins. The above data indicate that the binding affinity of copolymer film toward the two proteins could be easily tuned by adjusting the solvent polarity (i.e. CH_3_CN ratio (v/v)), which is beneficial for modulation of protein adsorption behaviours on the copolymer surface.

In addition, QCM-D experiment also provides an energy dissipation curve, which records real-time information about the variation in viscoelasticity and thickness of the copolymer ﬁlm. As shown in Figure 2(c), energy dissipation change on the copolymer film was negligible during the adsorption of α-casein or BSA (carrier solvent contained 10% CH_3_CN). By comparison, when the CH_3_CN ratio (v/v) was increased to 30%, the adsorption of both the two proteins induced a considerable increase in energy dissipation (see Figure 2(d), Δ*D*: 66 × 10^–6^ for α-casein and 44 × 10^–6^ for BSA). According to the classical QCM adsorption theory [31], this data demonstrated an evidential increase in viscoelasticity and thickness of the NIPAAm-*co*-ATBA ﬁlm after interaction with α-casein or BSA, suggesting that the copolymer chains might stretch into relaxed and swollen states. We speculated that the balance of intermolecular and intramolecular hydrogen bonds was disturbed by altering the solvent polarity, which might result in the conformational transition. The protein adsorption might in turn influence the conformational transition of the copolymer chains.

**Figure 3.**
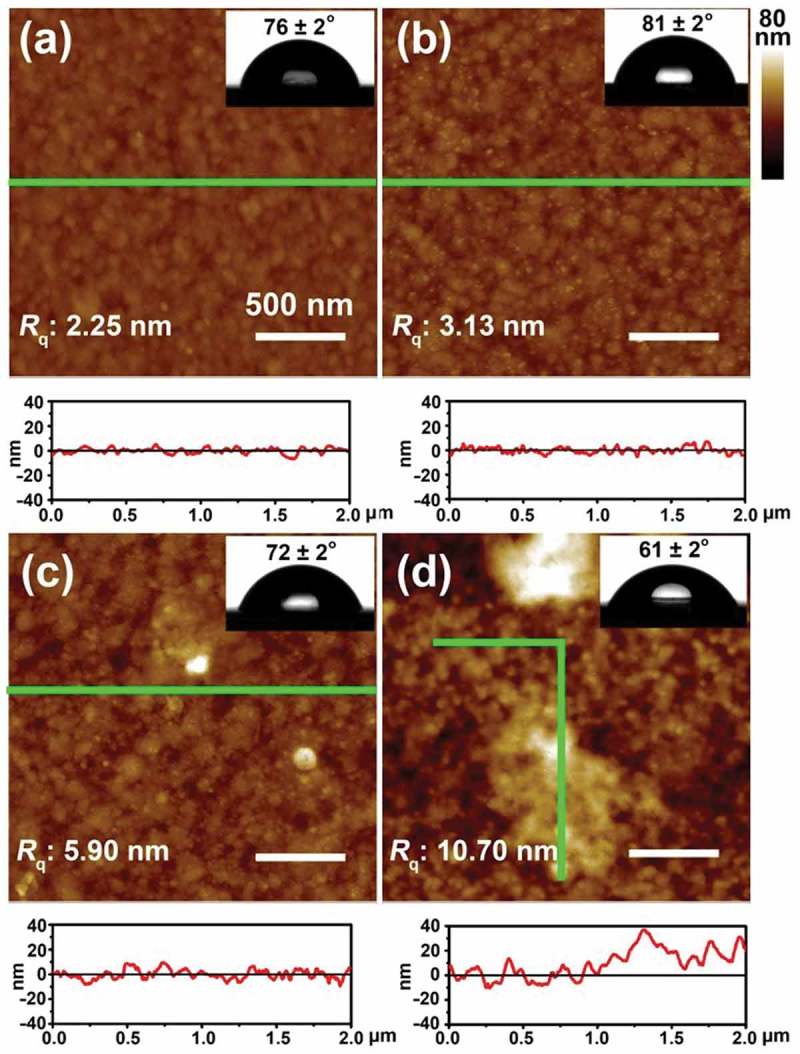
(a,b) AFM images of Au-coated QC resonator before (a) and after (b) modification with NIPAAm-*co-*ATBA copolymer thin film, and the corresponding section proﬁles along the green lines. (c,d) AFM images of NIPAAm-*co*-ATBA copolymer ﬁlm after being immersed in BSA (c) or α-casein (d) solutions (100 μg·mL^−1^ in CH_3_CN/H_2_O mixture with a volume ratio of 30:70) for 10 min at 20°C, and the corresponding section proﬁles along the green lines. Insets: water droplet profiles on the corresponding Au-coated QC resonator and copolymer surfaces.

This result was further confirmed by AFM investigation of morphological changes of the copolymer ﬁlm before and after interaction with α-casein or BSA [37]. Figure 3(a,b) displays the AFM images of Au-coated QC resonator before and after modification of the copolymer ﬁlm, in which no evidential changes in morphology and roughness were found. However, after being immersed in a protein solution (100 μg·mL^−1^ α-casein or BSA) for 10 min, the copolymer surface displays a clear morphology transition from a smooth state (Rq: 3.13 nm) to a notably rough state (Figure 3(c,d), Rq: 5.90 nm for BSA and 10.70 nm for α-casein). Especially in Figure 3(d), abundant swelling and expansion region were readily observed, indicating that the copolymer film expanded considerably after interaction with α-casein. Meanwhile, the copolymer surface wettability also changed from a hydrophobic state (CA: 81°) to a relatively hydrophilic state after interaction with the two proteins (CA: 72° for BSA and 61° for α-casein), as shown in the corresponding insets. On the basis of the QCM-D, AFM and CA data, we speculate that the copolymer chains may undergo globule-to-coil conformational transition, which originate from the flexible NIPAAm segments [27]. The combination of the NIPAAm segments enables the ATBA recognition units to hide or expose in the copolymer chain with the changes of solvent polarity. It will remarkably inﬂuence the binding and release of the phosphoproteins.

### 3.3. Fabrication and characterization of the NIPAAm-co-ATBA_0.35_@SiO_2_

**Figure 4.**
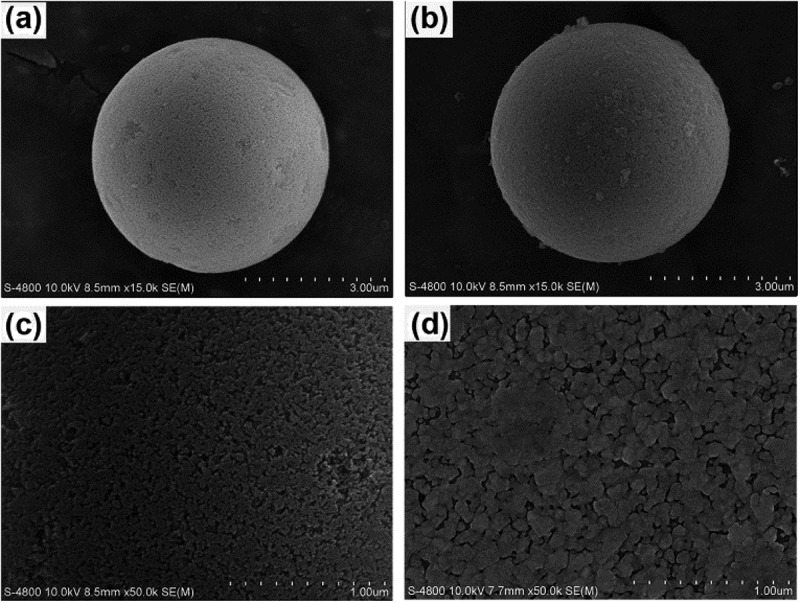
Scanning electron microscopy (SEM) images of porous silica microspheres before (a: ×15K, c: ×50K) and after (b: ×15K, d: ×50K) modification with NIPAAm-*co*-ATBA copolymer film.

**Figure 5.**
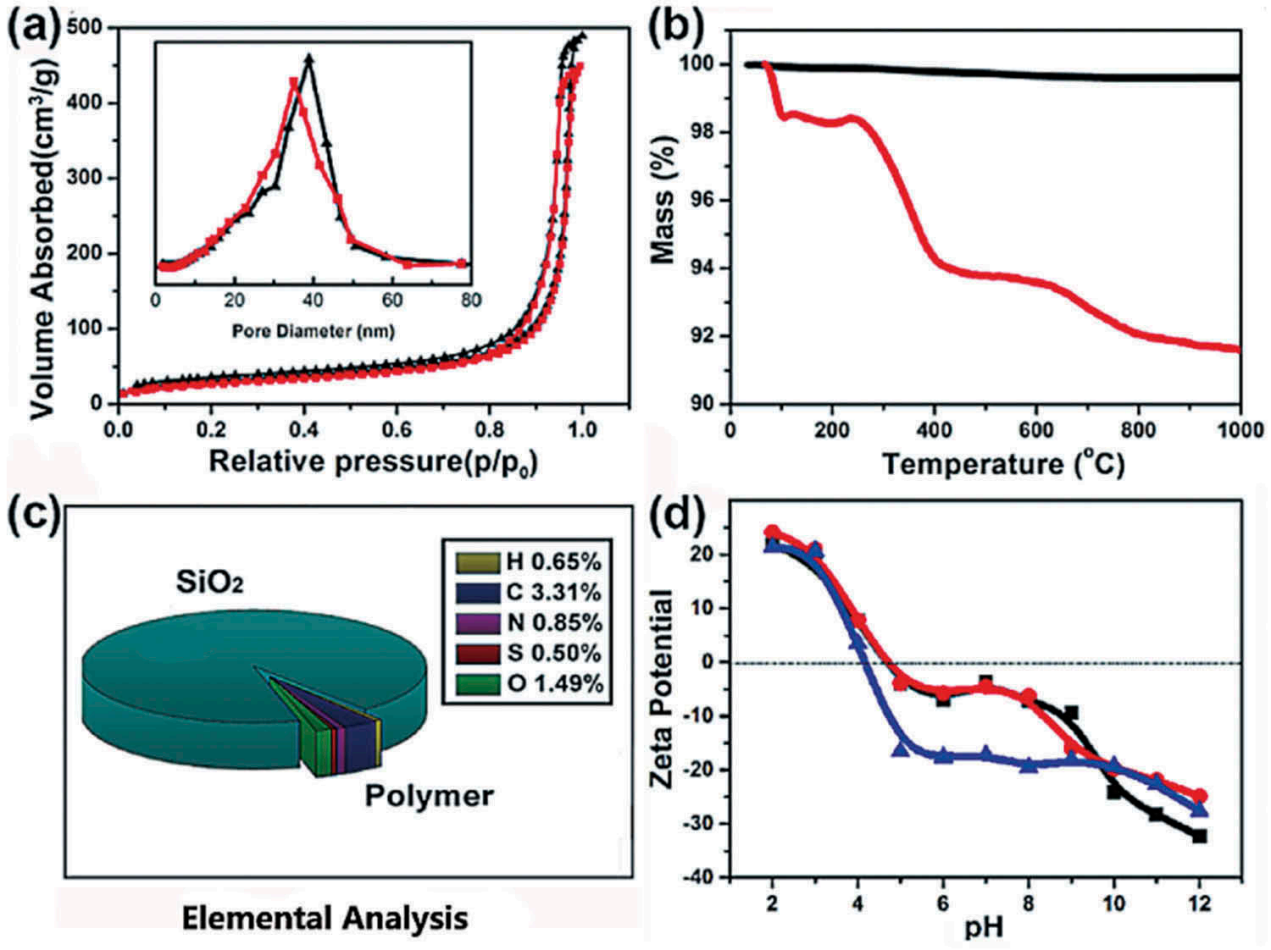
Characterization of the synthesized NIPAAm-*co*-ATBA_0.35_@SiO_2_. (a) Nitrogen adsorption and desorption isotherm curves of porous silica gels before (black) and after (red) modification with NIPAAm-*co*-ATBA_0.35_ copolymer; the inset shows the corresponding pore size distribution. (b) Thermal gravimetric analysis (TGA) curves of amino- (black) and NIPAAm-*co*-ATBA_0.35_ (red) modified silica gels. (c) Elemental analysis data of NIPAAm-*co*-ATBA_0.35_@SiO_2_. The oxygen content in the copolymer was calculated by combining the elemental analysis and TGA data; (d) PH-dependent zeta-potential change curves of NIPAAm-*co*-ATBA_0.35_@SiO_2_ before (black) and after complexation with BSA (red) or α-casein (blue).

Furthermore, for the fabrication of novel phosphoprotein enrichment material, the developed responsive copolymer was grafted onto the porous silica gel (diameter 5 μm, inner pore size 350 Å) through SI-ATRP [34]. The copolymer-modiﬁed silica gel (denoted as NIPAAm-*co*-ATBA_0.35_@SiO_2_) was characterized through scanning electron microscopy (SEM), nitrogen isothermal adsorption, thermal gravimetric analysis (TGA) and organic element analysis and zeta-potential analysis. The SEM images (Figure 4) demonstrated that the silica microspheres maintained its integrity and porosity after modification with copolymer film, and numerous polymer beads were clearly observed on the copolymer-modified microsphere surface. In combination with the nitrogen isothermal adsorption data and corresponding Brunauer-Emmet-Teller (BET) analysis (as shown in Figure 5(a), the inset illustrates the pore size distribution), the mesopores were also partially filled with copolymers, leading to a clear decrease in average pore size (approximately 3.8 nm) and in BET surface area (from 108.6 m^2^·g^–1^ to 93.2 m^2^·g^–1^). Then, the TGA data (Figure 5(b)) indicated that the grafting rate of the copolymer-modified silica gel was 6.8%, and the grafting density was calculated as 65.9 ng·cm^–2^ by combining the BET surface area data. Figure 5(c) illustrates the result of organic element analysis, which clearly shows the contents of diverse elements (i.e. C, H, N, and S) in the copolymer film on the silica microspheres. According to this data, the proportion of ATBA in the copolymer was calculated as 35%. These results suggested that NIPAAm-*co*-ATBA_0.35_ copolymer brush was successfully grafted onto the porous silica microspheres.

### 3.4. Enrichment of α-casein from the mixture of model proteins using NIPAAm-co-ATBA_0.35_@SiO_2_

For demonstration of the complexation of NIPAAm-*co*-ATBA_0.35_@SiO_2_ with α-casein or BSA, zeta-potential of the copolymer-modified silica surface was measured at different pH conditions. As shown in Figure 5(d), the zeta-potential of NIPAAm-*co*-ATBA_0.35_@SiO_2_ gradually decreased with the increasing pH values, indicating an obvious increase of anionic groups at alkaline conditions. This could be explained by the ionization of abundant carboxyl on the NIPAAm-*co*-ATBA_0.35_@SiO_2_ surface. Moreover, the zeta-potential remained stable (at –5 mV) in pH 6–8 before and after additions of BSA. Interestingly, by additions of α-casein, the stable zeta-potential plateau was remarkably decreased to –20 mV in a widened pH range of 5–10, suggesting that NIPAAm-*co*-ATBA_0.35_@SiO_2_ had strong binding affinity toward α-casein.

**Figure 6.**
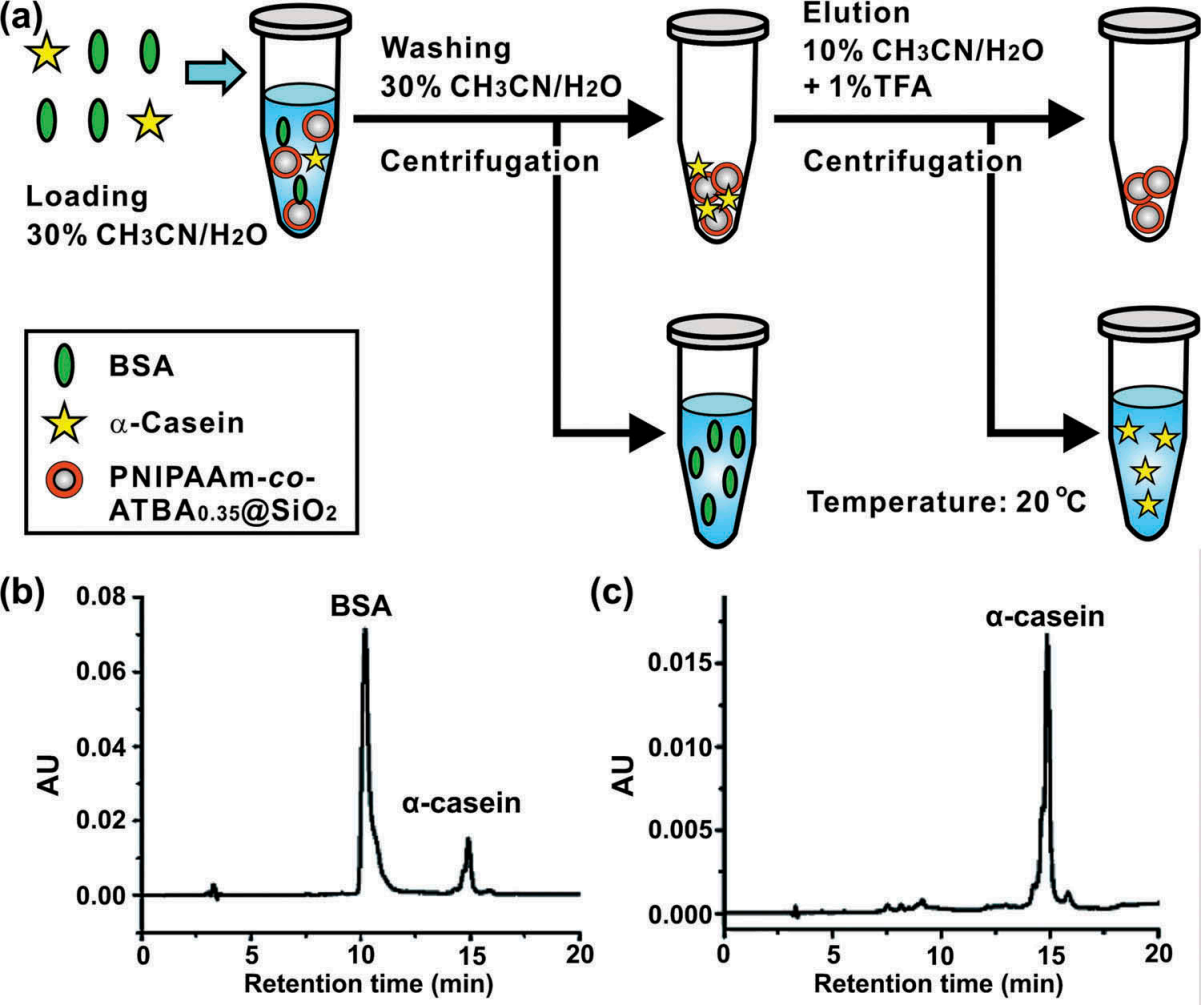
Enrichment and separation of phosphoprotein in model protein mixtures. (a) Schematic illustration of separation strategy based on a d-SPE mode. (b-c) HPLC chromatograms of BSA and α-casein mixture at a mass ratio of 5:1 before (b) and after (c) enrichment with NIPAAm-*co*-ATBA_0.35_@SiO_2._

Benefited from the distinct binding capacity of NIPAAm-*co*-ATBA_0.35_@SiO_2_ with α-casein or BSA, these copolymer-modified silica microspheres were applied to the separation of α-casein and BSA. Figure 6(a) illustrates the separation process in a dSPE mode [38,39]. Before treatment with NIPAAm-*co*-ATBA_0.35_@SiO_2_, the mixture of α-casein and BSA (mass ratio = 1:5) could be separated into two peaks in the chromatogram (Figure 6(b)). After the enrichment with NIPAAm-*co*-ATBA_0.35_@SiO_2_, only the phosphoprotein α-casein could be detected in the elution fraction (Figure 6(c)). The recovery was 28.2% by determining the peak areas of α-casein after and before enrichment with NIPAAm-*co*-ATBA_0.35_@SiO_2_.

### 3.5. Enrichment of phosphoproteins from defatted milk using NIPAAm-co-ATBA_0.35_@SiO_2_

**Figure 7.**
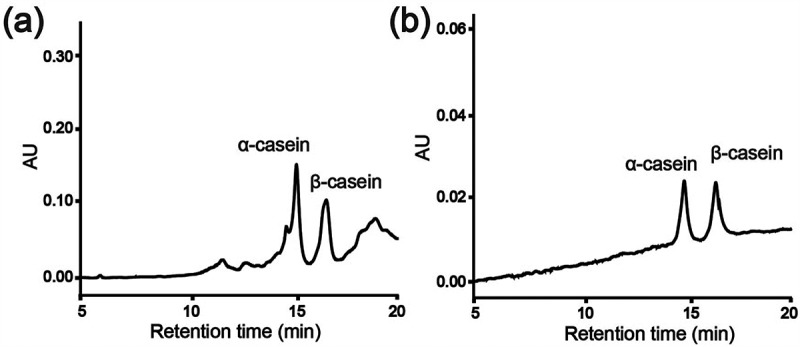
HPLC chromatograms of defatted milk before (a) and after (b) enrichment with NIPAAm-*co*-ATBA_0.35_@SiO_2._

To demonstrate the feasibility of NIPAAm-*co*-ATBA_0.35_@SiO_2_ in real samples, NIPAAm-*co*-ATBA_0.35_@SiO_2_ was used for selective enrichment of phosphoproteins from defatted milk. Before treatment with NIPAAm-*co*-ATBA_0.35_@SiO_2_, the defatted milk after precipitation contained various proteins and displayed many peaks in the chromatogram (Figure 7(a)). After the enrichment with NIPAAm-*co*-ATBA_0.35_@SiO_2_, only two peaks could be detected in the elution fraction (Figure 7(b)). After comparison with the standard samples, the two peaks were identified as belonging to the phosphoproteins α-casein and β-casein. These results suggested that our material had a good potential to enrich intact phosphoprotein from complex bio-samples.

## 4. Conclusions

In summary, our studies demonstrate the specific and tunable complexation of the well-designed ATBA receptor with α-casein driven by multiple hydrogen bonds. Benefited from the fundamentally different interaction features from conventional IMAC and metal oxide affinity chromatography (MOAC) materials [40,41], our smart polymer-based material realizes the separation of intact α-casein and BSA, which indicates the new opportunities of responsive polymer applied to phosphoprotein enrichment. However, there is still a huge gap between the synthetic smart polymers and natural recognizing proteins for the aspect of sensing accuracy and response speed, which may greatly influence the anti-interference capacity and efficiency of enrichment materials [42]. Therefore, further studies should be focused on the development of more specific recognizing receptors [43,44], and a highly reversible and expandable polymeric platform that allows a synergy among different copolymer components [45,46].
